# Enhanced HONO Formation from Aqueous Nitrate Photochemistry in the Presence of Marine Relevant Organics: Impact of Marine-Dissolved Organic Matter (m-DOM) Concentration on HONO Yields and Potential Synergistic Effects of Compounds within m-DOM

**DOI:** 10.1021/acsestair.4c00006

**Published:** 2024-04-30

**Authors:** Stephanie L. Mora García, Israel Gutierrez, Jillian V. Nguyen, Juan G. Navea, Vicki H. Grassian

**Affiliations:** † Department of Chemistry and Biochemistry, University of California San Diego, La Jolla 92037, California, United States; ‡ Department of Chemistry, Skidmore College, Saratoga Springs 12866, New York, United States

**Keywords:** nitrous acid, HONO, nitrate photochemistry, photosensitizer, hydroxyl radical scavenger, marine-dissolved organic matter, m-DOM, surface tension

## Abstract

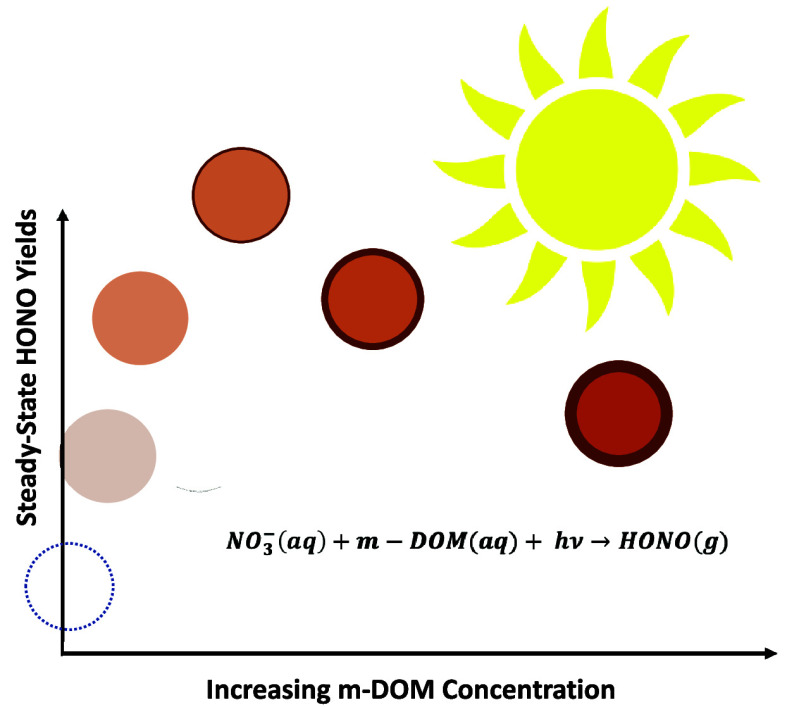

Nitrous acid (HONO) is a key molecule in the reactive nitrogen cycle. However, sources and sinks for HONO are not fully understood. Particulate nitrate photochemistry has been suggested to play a role in the formation of HONO in the marine boundary layer (MBL). Here we investigate the impact of marine relevant organic compounds on HONO formation from aqueous nitrate photochemistry. In particular, steady-state, gas-phase HONO yields were measured from irradiated nitrate solutions at low pH containing marine-dissolved organic matter (m-DOM). m-DOM induces a nonlinear increase in HONO yield across all concentrations compared to that for pure nitrate solutions, with rates of HONO formation increasing by up to 3-fold when m-DOM is present. Furthermore, to understand the potential synergistic effects that may occur within complex samples such as m-DOM, mixtures of chromophoric (light-absorbing) and aliphatic (non-light-absorbing) molecular proxies were utilized. In particular, mixtures of 4-benzoylbenzoic acid (4-BBA) and ethylene glycol (EG) in acidic aqueous solutions containing nitrate showed more HONO upon irradiation compared to solutions containing only one of the molecular proxies. This suggests that synergistic effects in the HONO formation can occur in complex organic samples. Atmospheric implications of the results presented here are discussed.

## Abstract 2

This study examines how marine-dissolved organic matter enhances the photochemical conversion of nitrate into nitrous acid and uncovers synergistic effects in the formation of nitrous acid in the environment.

## Introduction

The hydroxyl (•OH) radical is the most significant atmospheric oxidizer, as it reacts with trace tropospheric gases and it is critical in the formation of secondary organic aerosols including secondary marine aerosols.^1−6^ Despite its significance, •OH spatial and temporal information is still challenging to project by atmospheric models, mainly due to uncertainties in its sources.^7,8^ A major source of •OH in the troposphere is the photodissociation of nitrous acid, or HONO, which occurs at wavelengths between 300 to 400 nm.^9,10^ Over the last decade, several formation pathways of atmospheric HONO have been reported. These include direct emissions from soils,^11^ NO_2_ hydrolysis,^12,13^ NO_2_ reduction in the presence of photosensitizers,^14,15^ surface mediated reactions of nitrated surfaces,^16−19^ and aqueous and solid particulate nitrate photolysis.^20−25^ For nitrate photolysis, there are two reaction pathways, one leading to gas-phase NO_2_ and the other to HONO at low pH (and NO_2_^–^ at pH values greater than ca. 4).^20^ These reactions occur in the wavelength region near 300 nm within the tail of the solar spectrum.

Most studies over the last decade have focused on photochemical pathways for HONO formation from urban areas, ice sheets, and plant leaves.^26−32^ However, recently it has been suggested that the marine boundary layer (MBL) has significant daytime HONO, with the photoreduction of nitrate as a likely source.^21−23^ Additionally, despite the fact that HONO photodissociates within the solar actinic spectral region, its daytime concentration, especially in the MBL, has been found to reach quasi-steady-state levels, suggesting daytime photolytic sources.^21,22,25,33^ Recent field studies have found that nitrate photolysis reactions are likely the main contributor of photochemical HONO formation in the marine boundary layer (MBL).^21−23,25^ The source of nitrate in the MBL comes from the displacement of chloride with nitrate in aged sea spray aerosols due to heterogeneous chemistry with gas-phase nitrogen oxides (NO_2_, HNO_3_, and N_2_O_5_).^34,35^

Marine-dissolved organic matter (m-DOM), present in the acidic environment of sea spray aerosols (SSA), has been shown to enhance the formation of HONO in the MBL due to the presence of light-absorbing, photosensitizer molecular species.^36^ The photosensitized reduction of NO_2_ to HONO has been extensively investigated using humic acid as a chemically complex environmental photosensitizer.^14,15,20,37^ Importantly, studies combining computation and experiment have found that the UV–vis absorption of m-DOM falls within the actinic region and therefore opens up a potential photosensitization pathway due to m-DOM.^38−41^ Additionally, we have recently shown that broadband irradiation (λ > 280 nm) of aqueous nitrate solutions containing m-DOM, as well as solutions containing the known photosensitizer 4-benzoyl benzoic acid (4-BBA), enhance HONO formation by a factor of 5 and 3, respectively.^36^ The proposed mechanism involves the photosensitizer in its triplet state, abstracting hydrogen from water and producing hydroxyl radicals. These radicals subsequently react with aliphatic compounds, forming superoxide radicals. The superoxide radicals then react with NO_2_ or NO, byproducts of nitrate photolysis, resulting in nitrite formation. In acidic environments, such as those in SSA, nitrite is protonated to form HONO.^36^ Thus, the presence of non-light-absorbing, aliphatic compounds in complex m-DOM also play a role in enhanced HONO yields from nitrate photochemistry. The contributions of aliphatic and chromophoric components of m-DOM to HONO formation are relevant in SSA, given the higher concentration of marine organic species in SSA compared to bulk seawater due to bubble bursting mechanisms.^42−45^

Although these initial studies point to the role of m-DOM in enhancing HONO formation from nitrate photochemistry, questions remain on the effect of the m-DOM concentration on the yields of conversion of nitrate into HONO and whether synergistic effects due to the different compounds within m-DOM can play a role in enhancing HONO formation. To address the first question, we investigate the impact that m-DOM concentration has on the steady-state yields and the relative partitioning rates of gas-phase HONO from nitrate photochemistry in acidic, aqueous solutions. To address the second question, we have investigated HONO yields from irradiated nitrate solutions in the presence of mixtures of molecular proxies for different types of compounds, i.e., light-absorbing and non-light-absorbing, utilizing 4-BBA and EG, respectively. Our results show that HONO yields are enhanced from irradiated nitrate solutions containing the mixtures relative to the single components.

## Experimental Section

### Sample Preparation, Reaction Cell, and Solar Simulator

**Figure 1:**
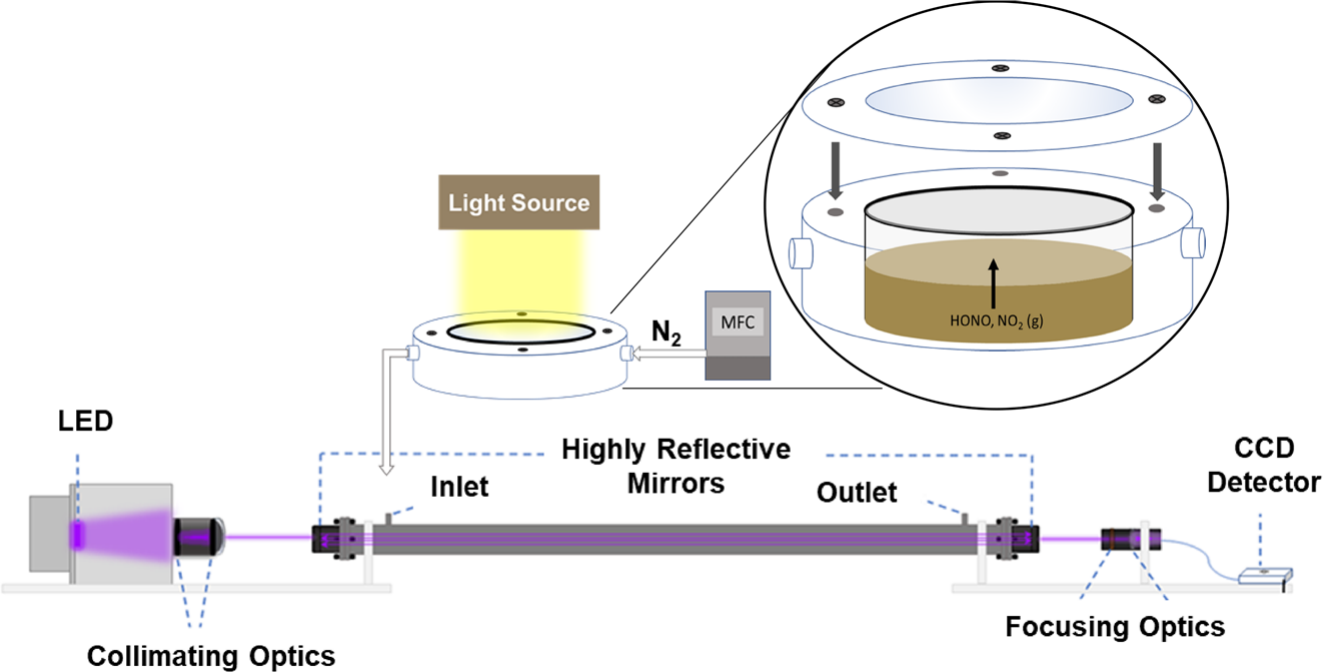
Schematic of the IBBCEAS used for NO_2_ and HONO detection. The aqueous sample cell is irradiated with a broadband Xe arc lamp solar simulator. A continuous flow of nitrogen carries the gaseous products from the reaction cell into the cavity. LED = light emitting diode, MFC = mass flow controller, CCD = charged coupled device.

The experimental setup and protocol have been previously described.^36^ Briefly, aqueous solutions containing 100 mM NaNO_3_ and varying amounts of m-DOM or mixtures of 4-BBA and EG were acidified using HCl. All irradiated m-DOM solutions were prepared from a 0.6 mg/mL stock solution. The stock solution was made by dissolving a known mass of dry m-DOM collected from the NSF-CAICE 2019 SeaSCAPE campaign^46^ in Milli-Q (MQ) water, rotating the mixture at room temperature overnight. For the solutions containing molecular proxies, 4-benzoyl benzoic acid from Sigma-Aldrich and ethylene glycol from Fisher Scientific were used for sample preparation. The mixture concentrations were chosen to be 1:3, 1:1, and 3:1 4-BBA to EG where the total organic concentration was 0.44 mM, as that is equivalent to 0.10 mg/mL 4-BBA. The desired amount of NaNO_3_ (Sigma-Aldrich) was added, and the solutions were acidified to pH 2.00 using 0.5 M HCl (Fisher Chemical). The aqueous solutions were then placed in a custom-made PTFE (polytetrafluoroethylene) reaction cell equipped with a quartz window to allow for sample irradiation. A broadband (λ > 280 nm) ozone-free 150 W xenon arc lamp (Newport Oriel) with an average output of 1000 Wm^–2^, equivalent to 1 sun irradiation intensity, was positioned above the reaction cell, as shown in Figure 1. A water optical filter removes infrared radiation to ensure isothermal 298 K reaction conditions. The irradiation time was 3 h, and a steady-state HONO concentration was reached ca. 2.5 h after the start of irradiation (*vide infra*). No significant changes in pH were found after the experiment.

### Measurements of Gas-Phase HONO and NO_2_

Gas-phase products, HONO and NO_2_, formed during the photochemical reaction were measured using a home-built Incoherent BroadBand Cavity Enhanced Absorption Spectrometer (IBBCEAS), as shown in Figure 1.^16,36^ The experimental setup gives an average effective pathlength (*P*_eff_) of 1.05 km across the spectral region from 360 to 390 nm with a maximum at the longer wavelengths of 1.57 km. The limits of detection for HONO and NO_2_ are 10 and 15 ppb, respectively. Before conducting the experiments, the experimental system, including the cavity, reaction cell, and all connecting tubing, is purged with N_2_ for at least 1 h. After the purge, data acquisition started for an hour under dark conditions, followed by the 3 h of irradiation. The rate of the inlet flow was set to 100 sccm into the IBBCEAS. Each spectrum is an average of 10 co-added scans with an integration time of 20 seconds for each, for a total of 200 seconds for each spectrum. Additional details of the data analysis are in the Supporting Information (SI) and include an example spectrum (Figure S1). The number of replicate experiments is discussed in the SI and Table S1.

**Figure 2:**
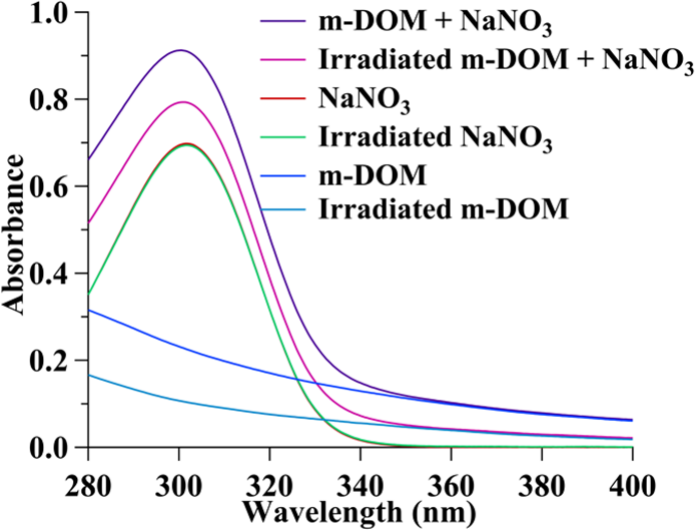
UV–vis absorption of a 100 mM sodium nitrate solution, 0.10 mg/mL m-DOM solution, and a solution containing both sodium nitrate and m-DOM before and after a 3 h irradiation period. All solutions were acidified to pH 2.00 before irradiation.

For other measurements, a surface tensiometer (AquaPi by Kibron) was used to measure the surface tension of the different solutions. An Agilent Cary 5000 UV–vis–NIR spectrophotometer was used to measure the absorbance of all solutions. For the samples containing m-DOM, spectra were taken before and after 3 h of irradiation to determine m-DOM optical changes due to irradiation. The absorbance spectrum of the irradiated sample was compared to that for non-irradiated solutions. Spectra were recorded for non-irradiated and irradiated solutions of 100 mM NaNO_3_, 0.10 mg/mL m-DOM, and 100 mM NaNO_3_ plus 0.10 mg/mL m-DOM, all acidified to pH 2.00 (see Figure 2). The UV–vis absorption spectra of 0.44 mM 4-BBA and 0.44 mM EG were also taken and are shown in Figure S2.

## Results and Discussion

### Characterization of Nitrate and m-DOM Solutions

The UV–vis absorption spectra of nitrate solutions with and without m-DOM show that irradiation with the solar simulator leads to a change in the optical properties of m-DOM only. The absorbance spectra of NaNO_3_ solutions before (red) and after (green) irradiation (Figure 2) show the characteristic absorption band with a λ_max_ at 305 nm corresponding to the *n* → π* transition.^20,47^ The UV–vis absorption spectra of these solutions show no significant change in the NO_3_^–^ peak intensity after 3 h of broadband irradiation. This implies that the concentration of nitrate is nearly constant with respect to the amount of HONO and NO_2_ being formed upon irradiation (*vide infra*) due to the small amount of depletion. In contrast, for solutions of m-DOM alone, the absorbance spectrum of m-DOM before (dark blue) compared with that after (light blue) irradiation shows a significant decrease in intensity. Similarly, for solutions containing both NO_3_^–^ and m-DOM, there is a continuous decrease in intensity across the 280 to 400 nm range of about 15% after irradiation, most likely due to changes in the absorption profile of m-DOM. Although this study focuses on the gas-phase production of HONO, it can be seen that the m-DOM itself is also changing. The changes in the absorption spectra of m-DOM alone shown in Figure 2 are likely a response to either direct photodegradation or the photooxidation process. Recent works suggest that triplet state m-DOM initiates oxidation involving •OH radicals, O(^3^P), or ^1^O_2_.^48−50^ In the presence of nitrates, this can lead to the formation of organonitrates, as has been previously observed by Ricker et al.^15^

In addition to UV–vis spectroscopy, surface tension measurements of different nitrate solutions with varying amounts of m-DOM (Table 1) were done to better understand the role of surface activity in the formation of HONO. The initial surface tension of the 100 mM NaNO_3_ solution in the absence of m-DOM was measured at 73.8 ± 0.1 mN/m. This value is higher than that of pure water, consistent with the behavior of salt-containing solutions, which exhibit higher surface tension values. As the concentration of m-DOM increases, the surface tension of the solution decreases from 72.3 ± 0.5 for a solution containing 0.01 mg/L m-DOM to 48.3 ± 0.1 for solutions containing 0.60 mg/mL m-DOM, showing that compounds within m-DOM are highly surface active and could impact partitioning of HONO into the gas phase.

**Table 1:** Surface Tension Measurements for the Solutions with Differing Amounts of m-DOM^a^

[m-DOM] (mg/mL)	surface tension (mN/m)
0.00	73.8 ± 0.1
0.01	72.3 ± 0.5
0.03	70.0 ± 0.5
0.05	66.6 ± 0.5
0.10	62.3 ± 0.5
0.20	50.9 ± 0.5
0.40	48.5 ± 0.3
0.60	48.3 ± 0.1

a The first row corresponds to a solution of 100 mM NaNO_3_ acidified to pH 2.00 with no m-DOM present. The uncertainty given is one standard deviation of triplicate measurements.

### HONO Formation from Nitrate Photochemistry in the Presence of Increasing m-DOM Concentrations

**Figure 3:**
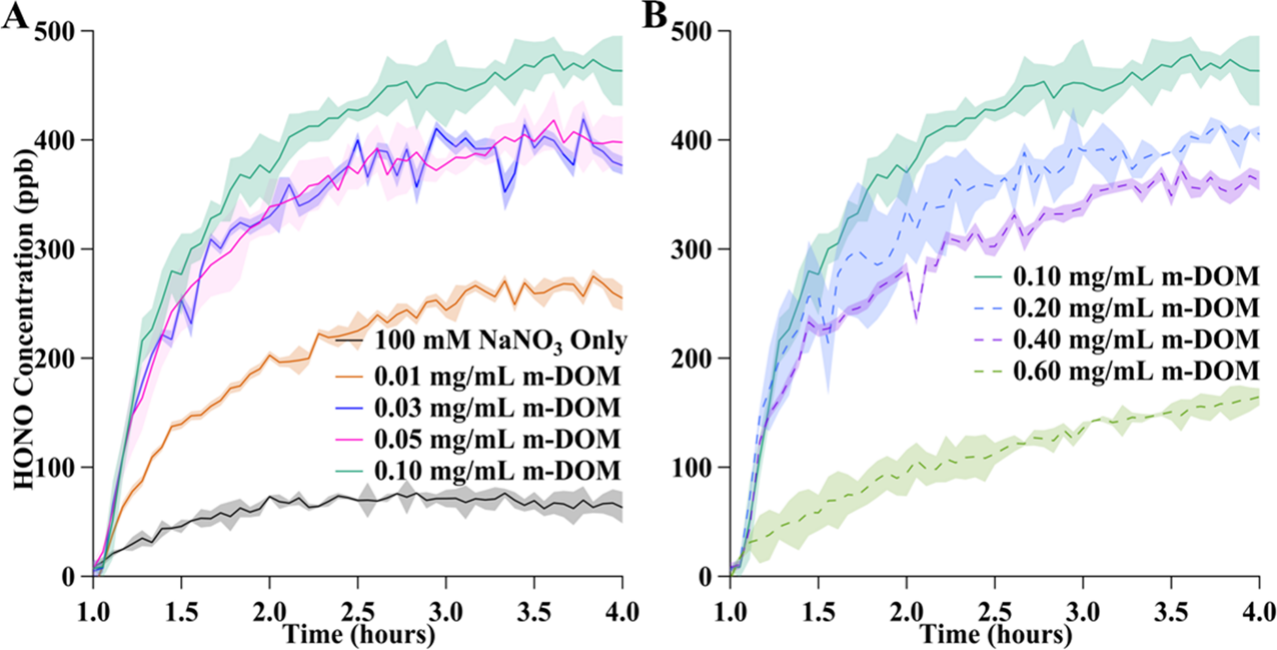
Temporal profiles of HONO formation from irradiation of nitrate solutions alone and with varying m-DOM concentrations. The graph shows only the period when the samples were being irradiated, which corresponds to *t* = 1 to 4 h with (A) concentrations of m-DOM of 0.0 to 0.10 mg/mL and (B) concentrations of m-DOM from 0.10 to 0.60 mg/mL. The solid lines correspond to solutions containing 0.00 to 0.10 mg/mL m-DOM, showing a monotonic increase in enhancement with m-DOM present, and the dashed lines correspond to the solutions containing 0.20 to 0.60 mg/mL m-DOM, which show a decrease with increasing m-DOM. Shading for each curve represents either one standard deviation or the error associated with the fitting of data to a reference HONO cross-section spectrum.

Solutions of 100 mM NaNO_3_ with varying amounts of m-DOM from 0.01 to 0.60 mg/mL were irradiated with a Xe arc broadband light source. During the irradiation time, gas-phase HONO and NO_2_ were detected by using IBBCEAS. Figure 3 shows time profiles for the formation of HONO from these different solutions. The data presented in Figures 3A and 3B both show the HONO yields during the 3 h period of irradiation with the solar simulator starting from time *t* = 1 h (for *t* < 1 h there was no irradiation). Figure 3A shows HONO measurements for the solutions containing only NaNO_3_ and NaNO_3_ plus m-DOM concentrations ranging from 0.01 to 0.10 mg/mL. Through this range of m-DOM mass concentrations, HONO was observed to monotonically increase with increasing m-DOM, with the highest value of HONO observed for the 0.10 mg/mL m-DOM sample. Experiments for solutions containing 0.20 to 0.60 mg/mL m-DOM, along with the 0.10 mg/mL sample shown for comparison, are plotted in Figure 3B and represented with dashed line traces. The data in Figure 3B show HONO levels decreasing with an increasing m-DOM mass concentration. The maximum measurements of HONO, [HONO]_max_, for all experiments occurred at the end of the 3 h irradiation period, which was the point where the majority of the experiments reached a pseudo steady state.

**Figure 4:**
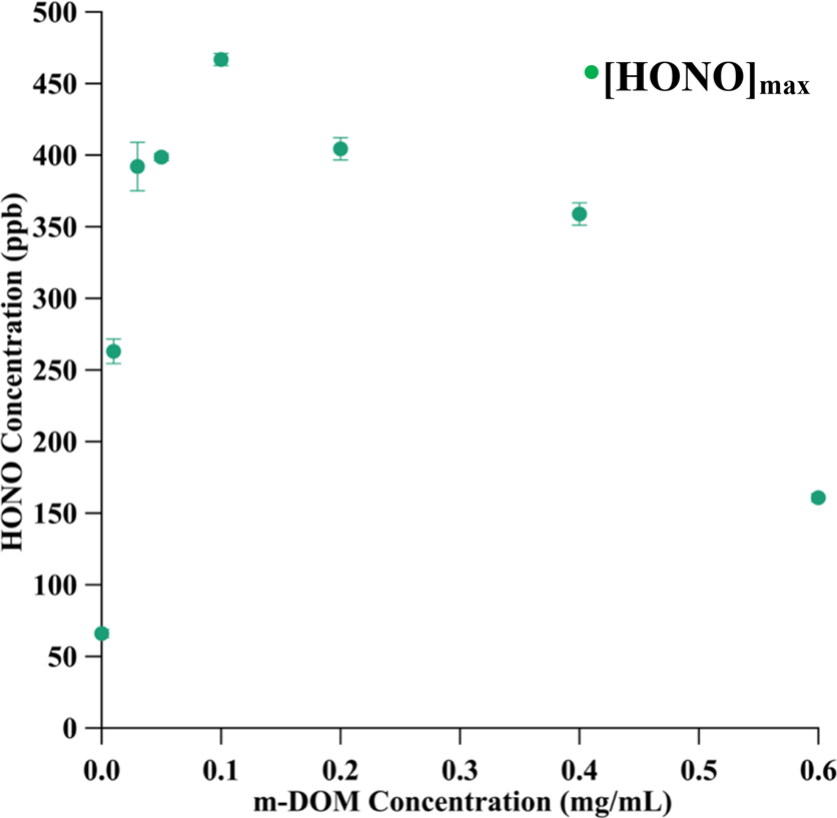
Maximum concentration of HONO in ppb ([HONO]_max_) measured from irradiating aqueous nitrate solutions as a function of m-DOM concentration. The error bars represent one standard deviation for the points that were taken to average the maximum HONO concentrations.

The maximum, steady-state concentration observed for HONO, [HONO]_max_, in experiments with varying concentrations of m-DOM indicate a complex dependence on m-DOM concentration. The solutions that only contain nitrate led to a [HONO]_max_ value of 66 ppb and all amounts of m-DOM lead to more than that, increasing with increasing m-DOM concentrations up to 0.10 mg/mL, where [HONO]_max_ peaked at 467 ppb. From 0.20 to 0.60 mg/mL, [HONO]_max_ decreased, with 0.60 mg/mL m-DOM having the lowest [HONO]_max_ at 161 ppb. These values are displayed in Figure 4 along with the data from the other concentrations. Interestingly, unlike HONO, the production of NO_2_ is not as impacted by the presence of m-DOM, and therefore, the HONO:NO_2_ ratio also increases when m-DOM is present. As detailed in previous work from our group,^36^ it is proposed that m-DOM enhances HONO formation by the combination of two pathways: the first pathway involves the reduction of nitrates into nitrites by a solvated electron formed by the charge splitting of excited m-DOM. At pH 2, nitrite can form HONO and partition into the gas phase via Reaction 3. The second pathway involves secondary reactions of superoxide radicals with photolysis byproducts NO_2_ and NO, leading to additional HONO formation and steady-state concentrations of NO_2_. The formation of the superoxide species can be photoenhanced by the formation of hydroxyl radicals from the irradiation of m-DOM. These enhancement pathways increase the levels of nitrite in the solution, leading to elevated HONO formation.^51^ Data for the NO_2_ profiles for all solutions is found in Figure S3.

**Figure 5:**
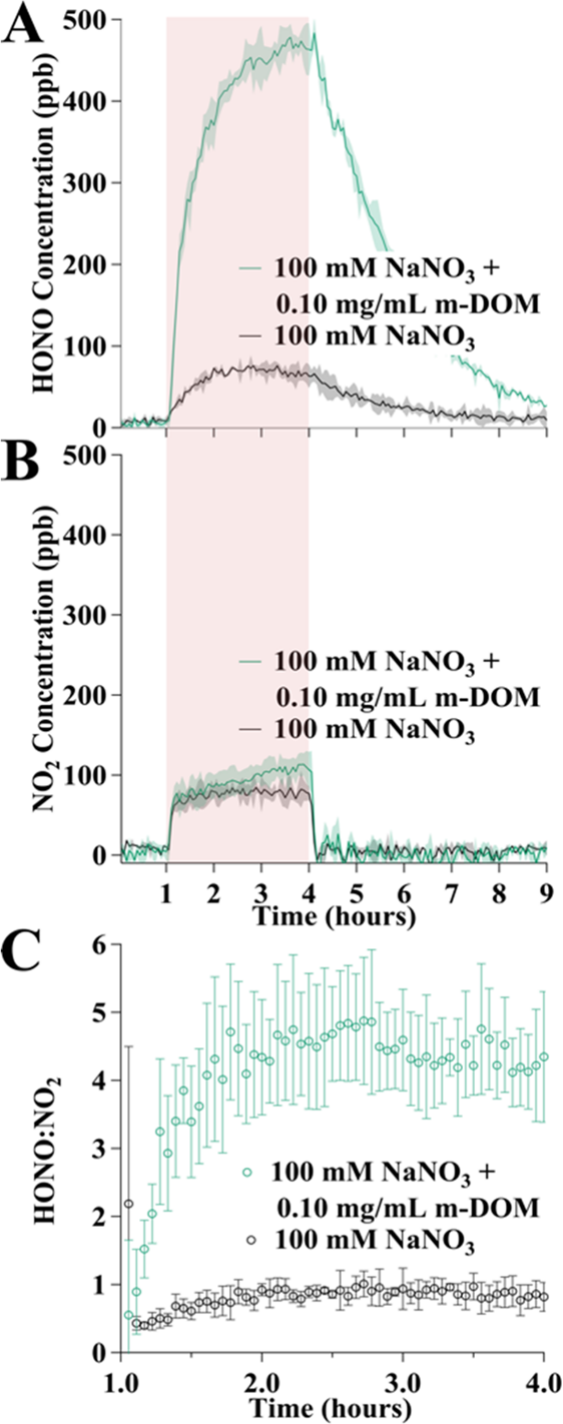
Time profile of gas-phase (A) HONO and (B) NO_2_ measured for solutions containing 100 mM NaNO_3_, black trace, and 100 mM NaNO_3_ + 0.1 mg/mL m-DOM, teal trace. The shading for each of the traces represents one standard deviation of triplicate experiments. The period of irradiation is denoted by the pink region (from time *t* = 1 to 4 h) in (A) and (B). (C) HONO:NO_2_ amounts for the same experiments during the period of irradiation reaching a maximum ca. 4.5 in the presence of m-DOM and 0.9 in the absence of m-DOM. The error bars represent error propagation for HONO and NO_2_.

Figure 5A shows HONO time profiles from 0 to 9 h for the experiments containing only nitrate and nitrate plus 0.10 mg/mL m-DOM. For these profiles, there is first a 1 h period before solutions are irradiated followed by a 3 h period of irradiation, denoted with the pink shading, and a 5 h period after the solar simulator is off. The HONO is enhanced up to 7 times at the end of the irradiation period. Figure 5B shows the time profiles for NO_2_ for the same experiments. In contrast to this substantial difference in HONO yields, NO_2_ concentrations did not change much in the presence of m-DOM. The lack of a significant difference in NO_2_ profiles with and without m-DOM is also the case for all m-DOM amounts except 0.60 mg/mL, which showed less NO_2_ by a factor of two (see Figure S3).

There are two major differences in the behavior of measured HONO and NO_2_ gas-phase concentrations from the irradiation of acidified nitrate solutions containing varying amounts of m-DOM compared to that of nitrate solutions alone. First, the time profiles of HONO and NO_2_ are significantly different. The rate at which NO_2_ reaches a steady state after the solar simulator is turned on and off is nearly immediate. In contrast, the formation of HONO takes longer to reach steady state during irradiation, and its decay rate after irradiation ends is also significantly slower compared to that of NO_2_. Second, once reaching steady state, the yield of NO_2_ shows less significant variations compared to HONO with varying amounts of m-DOM. The measurements of NO_2_ suggest rapid partitioning to the gas phase, whereas HONO partitioning is slower. These data can be attributed, in part, to the differences in the solubility of HONO and NO_2_ in water. Specifically, the Henry’s constant of NO_2_ compared to HONO is 2–3 orders of magnitude lower.^52,53^

Importantly, since HONO is significantly enhanced in the presence of m-DOM and NO_2_ is not, the HONO:NO_2_ ratios change with m-DOM concentrations. Aqueous nitrate photolysis has two product pathways, one that leads to NO_2_^–^ and O(^3^P) (ϕ = 0.001), and in the presence of excess protons, NO_2_^–^ forms HONO. The other product pathway forms NO_2_ and •OH (ϕ = 0.01).^54^ From these reactions, it is expected that the HONO:NO_2_ from solutions containing only NaNO_3_ would be near 0.1. However, Figure 5C shows that the experiments with only nitrate had a HONO:NO_2_ ratio of ca. 0.50 for the first 30 min of irradiation and then it approaches 0.9. The ratio being lower for the first 30 min is due to the slower formation rate of HONO compared to that of NO_2_. The observed HONO:NO_2_ ratio of 0.9 is higher than the previously observed ratio of 0.10. These higher HONO values are attributable to the acidification of the solutions to pH 2.00, which favors the protonation of nitrite with the concomitant formation of gaseous HONO.^20,36^ Additionally, because HONO partitions out of the solution phase into the gas phase, where the gas is then carried out of the reaction cell into the IBBCEAS with N_2_, there is no HONO equilibrium established between the solution and the gas phase. For the solution containing 0.10 mg/mL m-DOM, the HONO:NO_2_ ratio reached 4.5 during the course of the experiment. This HONO enhancement from marine relevant organics shows the HONO:NO_2_ nearly 5-fold in the marine boundary layer (MBL) is an important finding since the ratio HONO:NO_2_ is often a parameter in atmospheric models.^21−23^

Previously, we proposed enhanced formation of HONO (g) by m-DOM in irradiated NO_3_^–^ (aq) solutions via the formation of superoxide radicals from the reaction of hydroxyl radicals with organic aliphatic compounds, followed by secondary reactions with superoxide radicals with NO and NO_2_ leading to higher production of nitrite in the solution. This is further enhanced by the increase in hydroxyl radicals in the solution from the hydrogen abstraction of H_2_O with triplet excited state m-DOM.^36,54,56^ This process increases nitrite levels in the solution, resulting in elevated HONO formation at the low pH values observed in sea spray aerosol.^57^ This photoenhanced mechanism can be simplified as the photolysis of nitrates in the absence and presence of m-DOM:
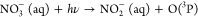
1
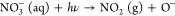
2

Reactions 1 and 2 represent the photolysis of aqueous NO_3_^–^ in the absence or presence of of m-DOM.^54^ The mechanism in the presence of m-DOM involves multiple reaction steps, resulting in the increased formation of HONO and the formation and consumption of NO_2_.^35^ The nitrite ion, NO_2_^–^ (aq)_,_ from Reaction 1 is protonated to form HONO (aq) at pH values below the pKa of HONO (pKa of 3.2),^20^ with the subsequent partitioning from the aqueous phase and into the gas phase, as shown below:
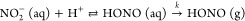
3

Under the experimental conditions, a continuous flow of nitrogen carries the gaseous HONO from the reaction cell into the IBBCEAS for detection away from the solution, which prevents the establishment of the equilibrium in Reaction 3. The formation of HONO (g) can be analyzed following pseudo-first-order kinetics driven by Reaction 3, with a generalized rate law:
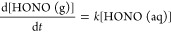
4

In the absence of m-DOM, the rate constant for the formation of HONO (g) is *k*, while *k*^m-DOM^ corresponds to the rate constants in the presence of m-DOM. The integrated rate law for the formation of HONO (g) can be expressed as
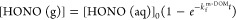
5where *k*_f_ denotes the photochemical formation rate constant of HONO as shown in Figure 3 from nitrate photolysis. The HONO data were fit to this expression, and the relative formation rates *k*_f_^m-DOM^/*k*_f_ were calculated. While the HONO yield increases for all solutions containing any amount of m-DOM, the *k*_f_^m-DOM^/*k*_f_ was less than 1 for all solutions (see Table 2), with statistically similar values at m-DOM concentrations between 0.03 and 0.4 mg/mL. This decrease in the *k*_f_^m-DOM^/*k*_f_ is due to the increase in the time to reach the steady-state concentration of HONO as m-DOM concentration increases up to 0.4 mg/mL. For the highest concentration, the relative formation rate was 0.3 ± 0.1. The *k*_f_^m-DOM^/*k*_f_ values from Table 2 are displayed in the SI along with the HONO concentration as a function of the surface tension values for the different m-DOM containing samples (Figures S4A and S4B). It is important to point out that the difference in the formation rates is not an artifact of HONO sticking to the walls of the carrier lines of the IBBCEAS cavity (see Figure S5).

**Table 2:** Relative Formation Rates of HONO from Solutions of Nitrate and m-DOM with Different m-DOM Concentrations^a^

[m-DOM] (mg/mL)	*k*_f_^m-DOM^/*k*_f_
0.00	1
0.01	0.54 ± 0.09
0.03	0.8 ± 0.1
0.05	0.76 ± 0.08
0.10	0.74 ± 0.08
0.20	0.8 ± 0.1
0.40	0.7 ± 0.1
0.60	0.3 ± 0.1

a Error represents the error in the fits to these curves.

After 3 h of irradiation, the broadband light was turned off with the concomitant decrease of HONO (g), as the partitioning to the gas phase. The partitioning of HONO (aq) into HONO (g) was fit to the first-order kinetics (eq 6) to obtain the rate constant for the decay of gaseous HONO. The fittings were done over HONO concentration after irradiation for nitrate solutions only, *k*_d_, and for nitrate solutions containing 0.10 mg/mL m-DOM, *k*_d_^m-DOM^.
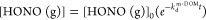
6

The decay rate constants do not differ from each other, with the solutions containing only nitrate and those containing 0.10 mg/mL m-DOM both leading to a *k*_d_/*k*_d_^m-DOM^ of 1. However, the surface activity of m-DOM in the aqueous nitrate solutions can explain the trends found in the HONO enhancement and observed formation rates. Evident in both the HONO formation profiles (Figure 3) and the [HONO]_max_ enhancement (Figure 4) with m-DOM present in the nitrate solutions, there is a trend where, with increasing m-DOM up to 0.10 mg/mL, the HONO enhancement increases and decreases past that point. This makes 0.10 mg/mL m-DOM present in 100 mM NaNO_3_ acidified solutions a turning point in the enhancement. Moreover, the observed formation rates of HONO are all lower than the rate of solutions only containing sodium nitrate, with a further decrease observed for the solutions containing the highest amounts amount of m-DOM.

Interestingly, the surface tension of the solutions, found in Table 1, also decreased with increasing m-DOM mass concentration. Solutions containing 100 mM NaNO_3_ had surface tension measurements of 73.8 ± 0.1 mN/m; the surface tension steadily decreased with increasing m-DOM reaching as low as 48.3 ± 0.1 mN/m for solutions having 0.60 mg/mL. This decrease in surface tension is due to the presence of surface active organic compounds present within m-DOM. This higher surface coverage of m-DOM can lower observed HONO due to m-DOM at the surface which can block the partitioning of HONO out of the solution, with the possible photolytic consumption of NO_2_^–^ (aq), leading to lower HONO formation rates at the highest concentrations.

This m-DOM sample was generally characterized to be rich in polycyclic, aromatic, and unsaturated compounds;^38^ these compounds will partition to the surface and be enriched at the air/water interface. The lack in change in HONO partitioning rates after exposure for solutions with only nitrate and nitrate plus 0.10 mg/mL m-DOM suggests a much more complex process, where competing surface effects and m-DOM enhancement factors need to be further considered.

Therefore, we propose that two competing effects are at play for m-DOM at higher mass concentrations. First, as the concentration of m-DOM increases, there is a corresponding rise in the level of HONO formation from the chemical enhancing mechanisms. However, as the concentration is further increased, m-DOM coverage at the air/water interface increases, causing the formation of a monolayer or more at the interface, resulting in a decrease in the partitioning of HONO from the solution into the gas phase. This effect would explain the decrease in [HONO]_max_ as well as the lower HONO formation rates for solutions containing high concentrations of m-DOM. This study builds on previous work that showed an enhancement of HONO in solutions containing m-DOM, where it was proposed to be due to both the secondary reactions of superoxide radicals with NO and NO_2_ as well as the increase in hydroxyl radicals from the hydrogen abstraction of water by a triplet state chromophore.^36^ In addition to these bulk solution enhancement mechanisms in HONO formation, the m-DOM surface coverage can also impact the partitioning of HONO. Furthermore, these m-DOM samples have high absorptivity, especially at higher concentrations, and the amount of light penetrating these solutions can decrease with increasing m-DOM concentration. In addition, for other environmental photosensitizers, such as brown carbon, it has been suggested that an increase in the concentration could result in higher rates of photooxidation or photodegradation. Analogously, higher concentration of m-DOM could hinder its photosensitizing capacity, with the concomitant decrease in the conversion of nitrates into HONO.^50,58^ Thus, overall, both physical and chemical effects play a role in the production of HONO from irradiated nitrate solutions containing m-DOM.

To further understand mechanisms of the enhancement, mixtures of molecular models, 4-benzoylbenzoic acid (4-BBA) and ethylene glycol (EG), for light-absorbing and non-light-absorbing components within m-DOM, respectively, were added to nitrate solutions and irradiated to determine if the enhancement of HONO in these mixtures was simply additive or if synergistic effects could play a role, as discussed below. The selection of the proxies is based on previous characterization of the chemical and optical properties of m-DOM, which shows absorbance by aromatic groups in the range between 280 to 500 nm, similar to that of 4-BBA.^41,59,60^

### HONO Formation from Nitrate Photochemistry in the Presence of Mixtures of 4-BBA and EG

**Figure 6:**
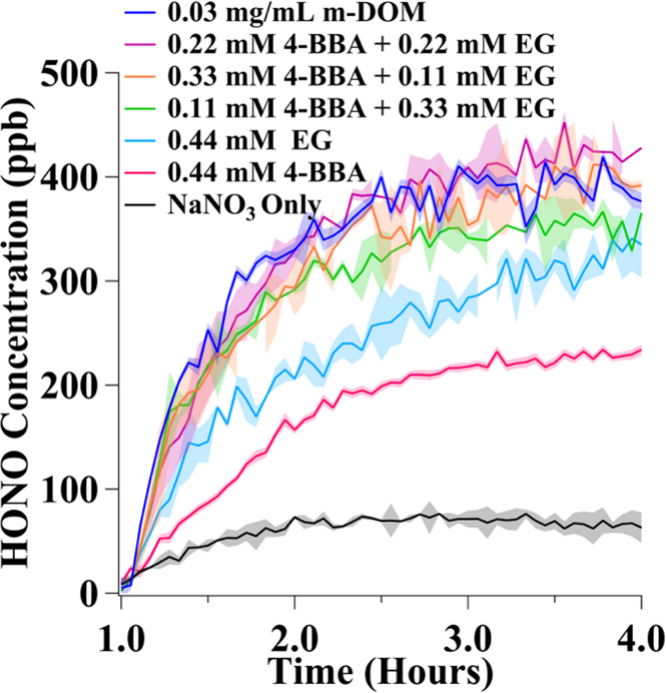
HONO time profiles in ppb experimentally measured during the period of irradiation (*t* = 1 to 4 h) with a Xe arc lamp of solutions containing 100 mM NaNO_3_ + 0.03 mg/mL m-DOM or 0.44 mM 4-BBA/EG, or varying mixtures of 4-BBA and EG with a total concentration of 0.44 mM.

Time profiles of HONO formation during the period of irradiation of solutions containing 100 mM NaNO_3_ with and without 4-BBA or EG as well as mixtures of 4-BBA and EG are shown in Figure 6. The mixtures of 4-BBA and EG were chosen so that the total organic concentration was equal to 0.44 mM in ratios of 1:3, 1:1, and 3:1, resulting in solutions containing 100 mM NaNO_3_ plus 0.11 mM 4-BBA and 0.33 mM EG (green trace), 0.22 mM 4-BBA and 0.22 mM EG (magenta trace), and 0.33 mM 4-BBA and 0.11 mM EG (orange trace), all acidified to pH 2.00 using HCl. 4-BBA was chosen as the light-absorbing molecular proxy for the chromophoric compounds in m-DOM,^41^ and EG was chosen as the non-light-absorbing molecular proxy for aliphatic compounds within m-DOM.^20^ The absorption spectra of 4-BBA and EG compared to m-DOM are shown in Figure S2.

Figure 6 shows the amount of HONO measured from these solutions in comparison to solutions containing 0.03 mg/mL m-DOM (darker blue trace), 0.44 mM 4-BBA in the pink trace, 0.44 mM EG in the lighter blue trace, and solutions containing no organics in the black trace. The 0.03 mg/mL m-DOM sample was chosen for comparison, as the solution resulted in surface tension values similar to the solutions containing the mixtures of 4-BBA and EG (see Table S2). Most interesting is the observation that the solutions containing mixtures of 4-BBA and EG containing 0.44 mM total organics led to more HONO compared to 0.44 mM of either of the individual compounds, suggesting synergistic effects play a role in enhanced HONO yields. In addition, the solutions containing 0.22 mM 4-BBA + 0.22 mM EG led to the most HONO compared to the other two mixtures. Compared to the solution containing 0.03 mg/mL m-DOM, the molecular proxy mixtures led to similar amounts of HONO and similar time course profiles. Evidently, mixtures containing 4-BBA and EG reproduce well the effect of m-DOM in HONO enhancement mechanisms. This is not a direct comparison as the exact compounds in this m-DOM sample and their abundance are unknown, but the molecular proxies were chosen to be broadly representative of the class of compounds found. Specifically, aromatic ketones such as 4-BBA and aliphatics such as EG glycol are some of the major compound types found in this m-DOM sample.^38^ Therefore, they can be used to investigate if the enhancement of HONO due to the photosensitization from light-absorbing compounds and secondary reactions from non-light-absorbing aliphatics in solution appear to be synergistic. Table S2 gives values of the surface tension for these solutions containing 4-BBA and EG. By comparing the HONO values to that resulting from the solution of 100 mM NaNO_3_ with 0.03 mg/mL m-DOM, HONO enhancement is significant, yet the effect of decreasing observed HONO enhancement due to the surface active compounds in m-DOM is not present. The NO_2_ profiles are provided in Figure S6.

**Figure 7:**
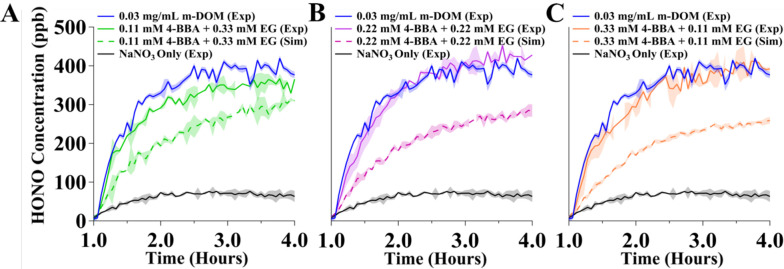
HONO concentrations in ppb (solid lines) measured from irradiation of solutions (*t* = 1 to 4 h)containing (A) 100 mM NaNO_3_ plus 0.11 mM 4-BBA and 0.33 mM EG, (B) 0.22 mM 4-BBA and 0.22 mM EG, and (C) 0.33 mM 4-BBA and 0.11 mM EG. Also shown in dashed lines are the simulated amounts for each experiment. The HONO irradiation profiles from solutions only containing 100 mM NaNO_3_ and 100 mM NaNO_3_ plus 0.03 mg/mL m-DOM are also shown for comparison. The shading represents one standard deviation for the experimental data for the experiments with only NaNO_3_ and the one containing m-DOM. The shading for the experimental mixture amounts is the error from fitting the data to a HONO reference spectra using DOASIS and error propagation for the simulated amounts.

Simulated amounts of HONO were calculated by assuming that the daytime enhancement of HONO formation is a linear combination of the contributions from the photosensitizing compounds (4-BBA) and the aliphatic compounds (EG). Thus, the simulated HONO profiles were calculated by first dividing each time point of the profiles for the experiments with 100 mM NaNO_3_ + 4-BBA and 100 mM NaNO_3_ + EG by 4. This division then would give the calculated intensity of 0.11 mM of each molecular proxy solution assuming linear behavior. At each time point, the mixture profiles were simulated by adding the amounts of each individual molecular proxy. For example, for the solution containing 0.11 mM 4-BBA and 0.33 mM EG, each time point was calculated by adding the HONO values at the respective timepoint (0.11 mM 4-BBA + 3 × 0.11 mM EG) to give the total HONO time point amounts. These simulated time profiles for each mixture are shown in Figure 7 in the dashed lines. If the enhancement of HONO due to photosensitizing compounds (4-BBA) and aliphatic compounds (EG) was linearly additive, then this is what the expected HONO time profiles would look like for the solutions containing the mixture of the two compounds.

From these simulations, it can be seen that HONO formation profiles for the mixtures calculated this way were lower than the experimental amounts measured. This suggests that there are synergistic effects occurring with these different compounds. In an environment where both photosensitizers with relatively limited photooxidation and aliphatic compounds are present, the two compound types synergistically enhance the photolysis of nitrates leading to HONO. Furthermore, the experiments with 0.22 mM 4-BBA + 0.22 mM EG and 0.33 mM 4-BBA + 0.11 mM EG led to the highest amount of HONO and had a higher difference due to the simulated amounts, signifying that the synergism in the HONO enhancement is greater when there is equal to or greater concentration of 4-BBA than EG in the solutions. This synergistic enhancement from more photosensitizing molecules in the solution is not likely due to the photosensitized reduction of NO_2_ to HONO because the measured amounts of NO_2_ are not statistically different between the different mixture experiments. The NO_2_ profiles are provided in Figure S6. The more likely reasoning is that the photosensitizer 4-BBA abstracts a hydrogen from water when it is excited in the triplet state.^56^ This leads to an increase in hydroxyl radicals, which react with aliphatic compounds like ethylene glycol to form superoxide radicals via a non-photochemical pathway.^59^ These superoxide radicals react with NO and NO_2_ in the solution, increasing HONO levels by enhancing nitrite formation in an acidic aqueous solution:^36,55^
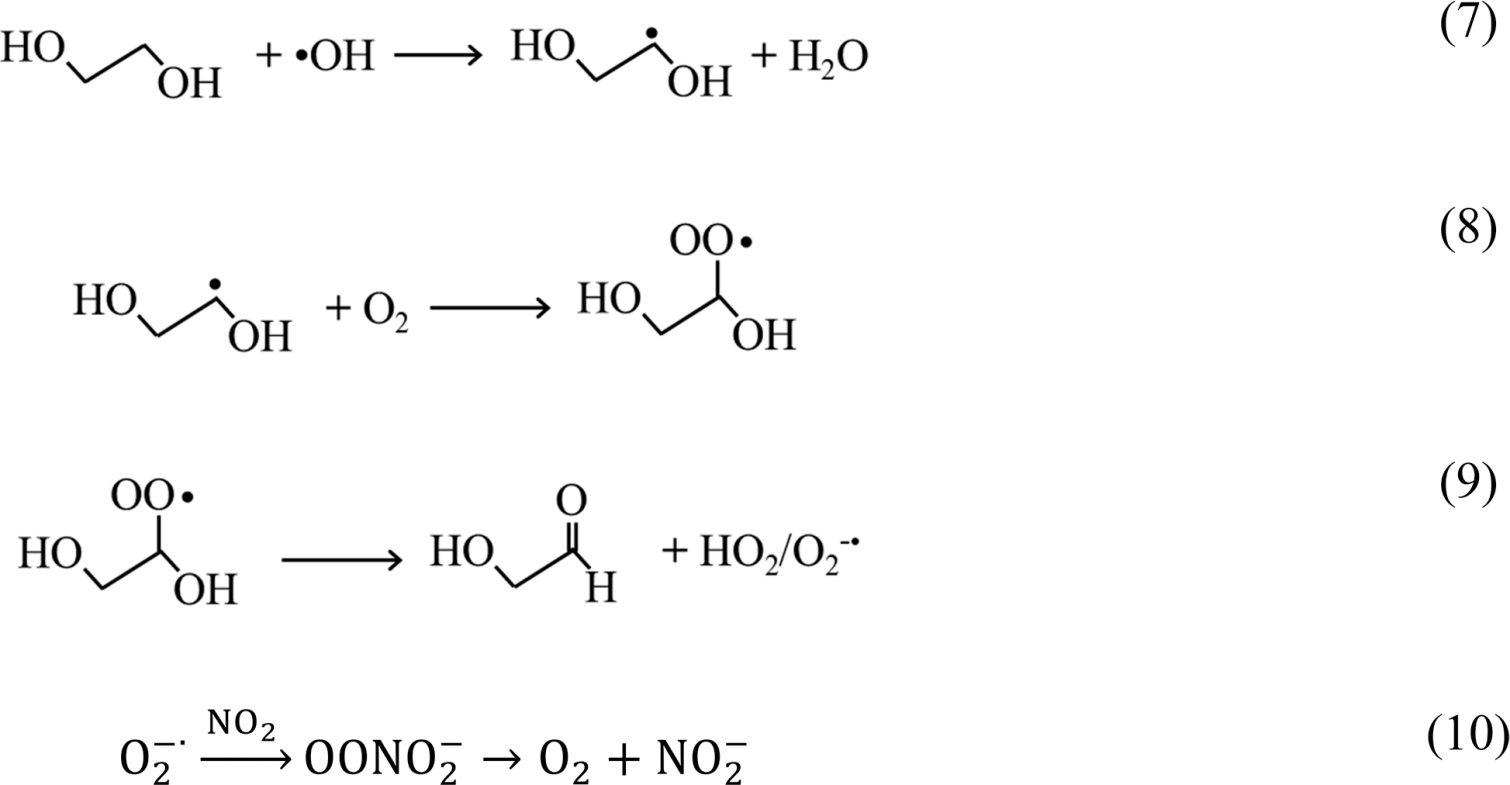
7

In our earlier publication, we proposed that HONO enhancement due to m-DOM in the solutions was due to both aliphatic and photosensitizing compounds in the sample.^36^ This finding not only confirms that these mechanisms are responsible for the enhancement but also gives insight into its synergistic nature. This understanding is crucial when assessing this effect in environmental samples like m-DOM and other organics within SSA, which include various compounds acting as photosensitizers or aliphatic substances.^38,42,62^

## Conclusion and Atmospheric Implications

The effect of varying the amounts of m-DOM present in acidic aqueous nitrate solutions on the photochemical formation of HONO was quantified. Experimental variables presented herein were designed to mimic conditions observed in SSA, such as acidic pH up to 2.00 for submicron SSA^57^ and m-DOM collected from the NSF-CAICE 2019 SeaSCAPE campaign.^46^ Overall, the presence of m-DOM increased HONO yields across the concentrations investigated compared with HONO formation from nitrate solutions in the absence of m-DOM. Interestingly, the increase was not linear or monotonic across the m-DOM mass concentrations investigated. At the lower mass concentrations of m-DOM (0.01–0.10 mg/mL), the yields of HONO increased with increasing m-DOM concentration, leading to a maximum steady-state concentration of 467 ppb compared to only 66 ppb for solutions containing only nitrate. At concentrations above 0.10 mg/mL m-DOM, increasing the m-DOM concentration led to a decrease in HONO yields with the highest concentration, 0.60 mg/mL m-DOM, giving a steady-state HONO concentration of 161 ppb. While HONO yields increased, there was no similar increase in photochemical production of NO_2_ from nitrate photochemistry. This led to an increase in the HONO:NO_2_ ratio by approximately five-fold in the presence of 0.10 mg/mL m-DOM. Additionally, the relative rates of HONO partitioning into the gas phase decreased with m-DOM present at high concentrations correlating to a decrease in the surface tension of the solutions, which impacts HONO partitioning into the gas phase. Overall, this study sheds light on the daytime HONO formation through m-DOM photosensitization of nitrates. Yet, the role of chloride ions in natural settings should be considered given recent findings.^15,61,63,64^

We also investigated if HONO enhancements in the presence of m-DOM from the photochemistry of aqueous nitrate solutions are due to additive or synergistic enhancement from the different types of compounds in m-DOM. Chromophoric and aliphatic compounds within m-DOM led to HONO enhancement via two different mechanisms; thus, using molecular model compounds can give insight into the specific mechanisms occurring. In particular, m-DOM absorbance in the 280–500 nm region, attributed to the π → π* transition from aromatic groups,^39^ is similar to that of 4-BBA.^41,59,60^ In addition, 4-BBA keto and carboxylic acid groups, along with its speciation in the aqueous phase, mimic m-DOM chromophores, while EG is used as an aliphatic diol moiety. While surface effects may vary, the mimics offer insights into the molecular mechanisms underlying HONO formation through photosensitized processes.

For these experiments, acidic aqueous nitrate solutions in the presence of mixtures of 4-BBA and EG containing a total of 0.44 mM organics led to more HONO enhancement (between 350 and 400 ppb) than 0.44 mM of either of the molecular proxies on their own. We propose that this signifying HONO enhancement from a mixture of chromophoric and aliphatic compounds in nitrate solutions is synergistic. Additionally, when comparing these experimental results to simulated amounts of what the solutions would be from a theoretical linear addition of HONO formation from the individual proxies, the experimental measurements were much higher; supporting the idea of a synergistic enhancement when there is a mixture of organics in the solutions. The solutions with 1:1 and 3:1 4-BBA to EG led to the highest steady-state yields of HONO.

Finally, although the enhancement of photolysis due to particulate nitrate compared to that of nitric acid has been suggested for the higher-than-expected nitrate to HONO amounts, the enhancement due to marine organics present in the environment has not. This study has provided a concentration dependence of marine relevant organics and synergism effects on the enhancement of HONO that must be considered in MBL conditions. Though the total organic concentration in SSA is unclear, it is known that in ultrafine SSA, the composition is dominated by organic carbon (OC).^42−44^ This study provides a range of what would likely be a high OC ratio SSA range for organic enhancement of HONO formation from nitrate photolysis in acidic SSA.^57^

## Supporting Information Available

A description of data analysis; UV–vis spectra of m-DOM, 4BBA, and EG; NO_2_ concentration profiles for various m-DOM concentrations and proxies; steady-state HONO concentration with respect of surface tension and m-DOM concentration; HONO evacuation profiles from the cavity; and m-DOM extraction details and replication of experiments with limited amount of m-DOM (PDF)
