# Supplementary material for: Enhanced HONO Formation from Aqueous Nitrate Photochemistry in the Presence of Marine Relevant Organics: Impact of Marine-Dissolved Organic Matter (m-DOM) Concentration on HONO Yields and Potential Synergistic Effects of Compounds within m-DOM
Source: ACS EST Air. 2024 Apr 30;1(6):525–35. doi: 10.1021/acsestair.4c00006 (PMC11184552; doi:10.1021/acsestair.4c00006)
Supplement: Supplementary file 1 — ea4c00006_si_001.pdf [file ea4c00006_si_001.pdf]

Supporting Information:

Enhanced HONO formation from aqueous nitrate photochemistry in the presence of marine relevant organics: Impact of marine dissolved organic matter (m-DOM) concentration on HONO yields and potential synergistic effects of compounds within m-DOM

Stephanie L. Mora García,[a] Israel Gutierrez[a], Jillian V. Nguyen,[a]

Juan G. Navea,[b*] and Vicki H. Grassian[a*]

[a]Department of Chemistry and Biochemistry, University of California San Diego, La Jolla CA 92037

[b]Department of Chemistry, Skidmore College, Saratoga Springs, NY 12866

[*]Authors to whom correspondence may be addressed. Email:vhgrassian@ucsd.edu and

The supporting information document contains 10 pages total with 6 figures and 2 tables.

Data Analysis

The DOASIS analysis software for differential optical spectroscopy was used to deconvolute the data acquired by the IBBCEAS.[1] The literature HONO and NO_2 absorption cross sections were convoluted to match the resolution of the detector used.[2,3] The software deconvolutes the acquired spectra by fitting the data to these literature values. The rest of the data is fit to a polynomial containing non-HONO absorption and Mie and Rayleigh scattering or a residual trace for data not fitted to the reference spectra or the polynomial. This is shown in Figure S1 for a solution of 100 mM $NaNO_3$ at pH 2.00.

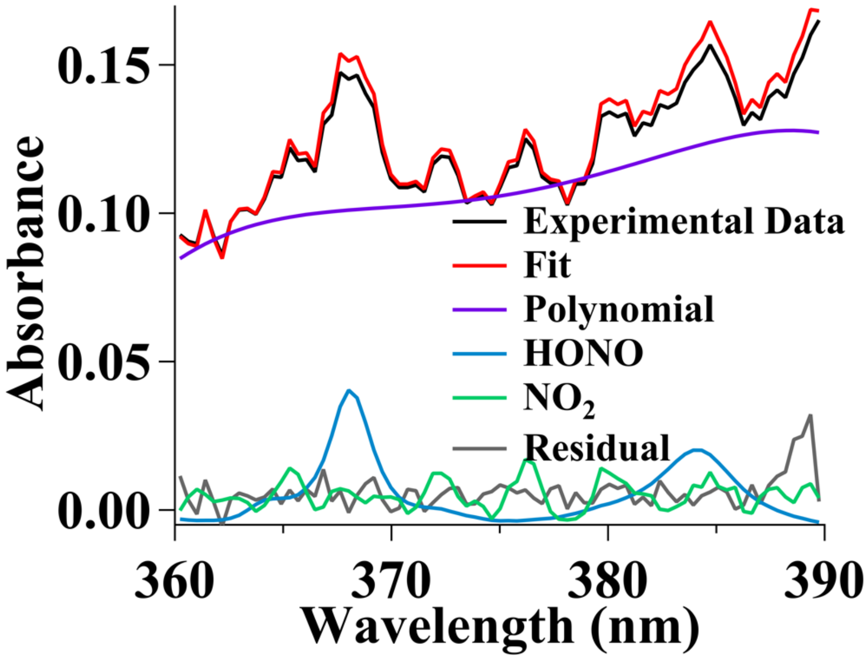

Figure S1. The experimental spectrum (black trace) is fit by DOASIS (red trace) and then separated into the absorbance due to HONO (blue trace) and NO_2 (green trace) by fitting them to literature HONO and NO_2 spectra. Also displayed are the absorbance due to Mie and Rayleigh scattering and any other non-HONO and NO_2 absorptions fitted to a polynomial (purple trace) and any wavelength dependent residual absorption data not able to be fitted (gray trace). This is the deconvolution of one spectrum for an experiment of 100 mM $NaNO_3$ at pH 2.

UV-Vis Absorption Spectroscopy of Molecular Proxies

The absorbance spectra of the molecular proxies used in this study was taken and compared to m-DOM. The range of absorption was 280-500 nm as this is the range of absorption of m-DOM that overlaps with the solar spectrum.[4,5] Figure S2 displays 0.03 mg/mL m-DOM in dark blue having a broad absorption profile in the entire range. The absorbance from m-DOM in this region corresponds to the $\pi \rightarrow \pi^*$ transition corresponding to the aromatic groups in m-DOM.[4] The absorption of 0.44 mM 4-BBA (pink) has a peak not shown in Figure S3 at 260 nm, but the tail of this absorption is shown and correspond to the both the $n \rightarrow \pi^*$ and $\pi \rightarrow \pi^*$transitions.[6] Lastly, 0.44mM EG (light blue) does not absorb light in this region, having some absorption starting at energies higher than 280 nm and peaking at 200 nm, which is higher energy than relevant in this study, making it a viable non-light absorbing marine boundary layer aliphatic proxy.[7]

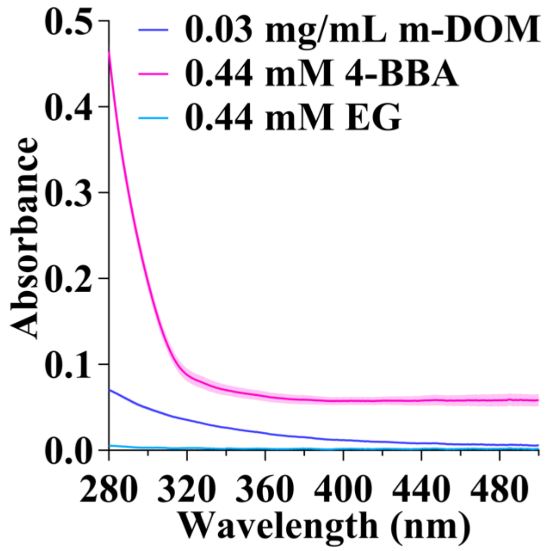

Figure S2. The absorption of the molecular proxies 4-BBA and EG both at 0.44 mM in concentration. These are compared to 0.03 mg/mL of m-DOM all solutions contain only the organics in MQ water acidified to pH 2.0 with HCl. The shading corresponds to the error in the form of one standard deviation from triplicate measurements. In some cases the shading is not evident as the error is smaller than the width of the line.

NO_2 Profiles of Irradiated Nitrate Solutions Containing m-DOM

The profiles of NO_2 yields measured for experiments containing only 100 mM $NaNO_3$ and 100 mM $NaNO_3$ plus varying m-DOM mass concentrations in mg/mL during the period of irradiation are shown in Figure S3. Unlike the HONO formation profiles shown in the main text, NO_2 reached steady state almost immediately. Also different from HONO, NO_2 concentrations did not vary with a dependence on m-DOM concentration. The lack of enhancement in NO_2 formation from m-DOM supports the mechanisms suggested as they only enhance HONO and not NO_2 as well. The only m-DOM mass concentration that led to a significantly different NO_2 concentration was that of 0.60 mg/mL m-DOM. This supports the role of m-DOM at the surface decreasing the partitioning abilities of HONO and NO_2 out of the solution.[8] Though the effect is more visible for HONO than NO_2 due to their gas solubility abilities in water.[9,10]

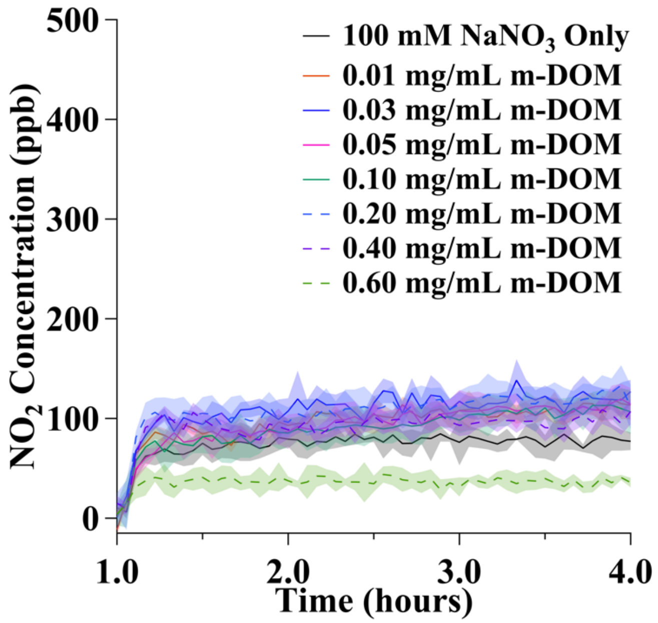

Figure S3. The NO_2 concentration measured in ppb during the irradiation period of the experiments (t = 1 – 4 hours) for all solutions containing acidified 100 mM $NaNO_3$ with varying amounts of m-DOM. The dashed traces correspond to the solutions that led to less HONO enhancement with increasing m-DOM concentration.

HONO Formation

The maximum, steady-state concentration observed for HONO, $[HONO]_{max}$, in experiments with varying surface tension. Similarly, the relative rate constant was from Table 2 was plotted with respect of the concentration of m-DOM.

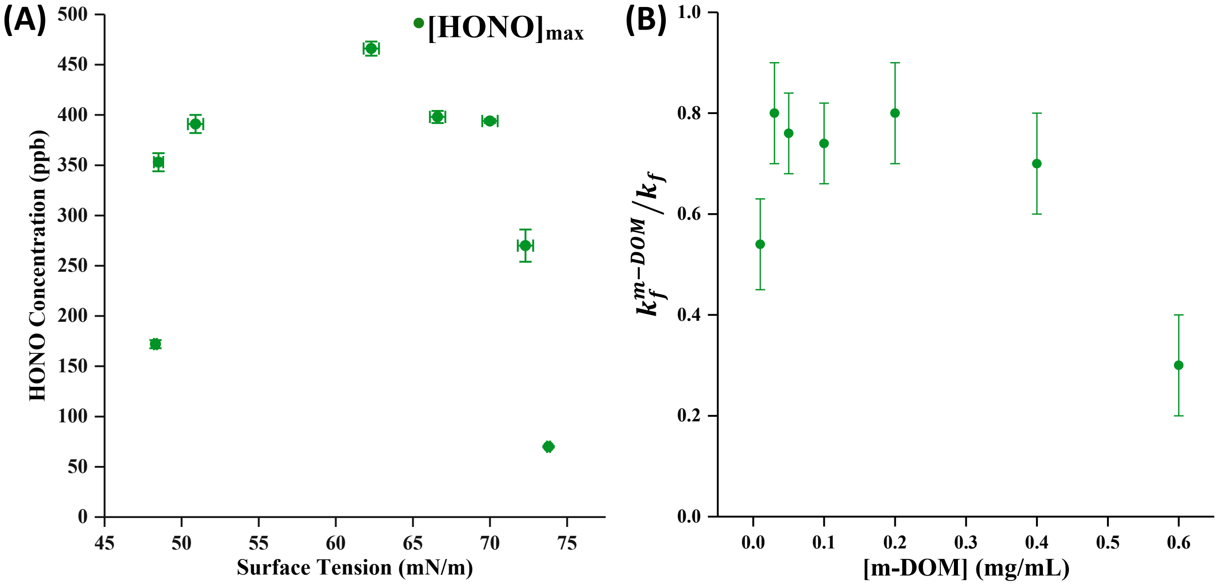

Figure S4. (A) Maximum concentration of HONO in ppb ($[HONO]_{max}$) measured from irradiating aqueous nitrate solutions as a function of surface tension. The error bars represent one standard deviation. (B) Relative formation rates of HONO from solutions of nitrate and m-DOM with different m-DOM concentrations to HONO from solutions of with nitrate only.

HONO Profiles of Irradiated Nitrate Solutions Containing m-DOM

The HONO formation profiles for all solutions containing m-DOM are shown in Figure 3 and the decay profiles for solutions containing only nitrate and nitrate plus 0.10 mg/mL m-DOM are shown in Figure 5 of the main text. A control experiment was performed where solutions containing 100 mM $NaNO_3$ plus 0.10 mg/mL m-DOM was exposed to light from the Xe-Arc lamp solar simulator for three hours and the decay was measured by only turning off the lamp. This same experiment was done where the lamp was turned off as well as the path of the carrier gas from the reaction cell into the IBBCEAS was cutoff while keeping the rate of carrier gas the same. Figure S5 below shows that evacuation of HONO from the cavity happens within 30 minutes of cutting off the path from the reaction cell with the majority happening within the first 15 minutes. In contrast, keeping the carrier gas flowing over the reaction cell leads to a HONO decay profile that requires more than 5 hours to no longer detect HONO. Therefore, the profiles observed are indicative of kinetics of the gases partitioning into the gas and not HONO adsorbing to the walls of the experimental setup.

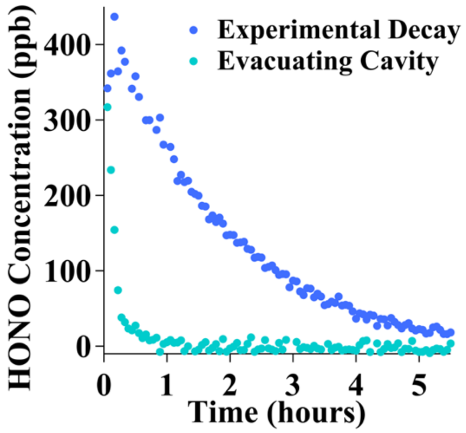

Figure S5. The decay (royal blue) or evacuation (cyan) of HONO was measured in the experimental set up. Time = 0 is the point where the acidified to pH 2.0 solutions of 100 mM $NaNO_3$ + 0.10 mg/mL m-DOM where no longer exposed to the solar simulator.

NO_2 Profiles of Irradiated Nitrate Solutions Containing 4-BBA and EG

The NO_2 measurements for the experiments containing nitrate and mixtures of 4-BBA and EG did not differ much from each other. Figure S6 shows the measured NO_2 measured during the period of irradiation for the 4-BBA and EG mixture solutions experiments as well as solutions containing individual compounds and 0.03 mg/mL m-DOM. All solutions led to NO_2 between 60 - 90 ppb for the entirety of the irradiation period, with the solutions containing 0.44 mM 4-BBA led to slight less averaging at 50 ppb and the solution with 0.03 mg/mL m-DOM led to slightly more averaging at 100 ppb.

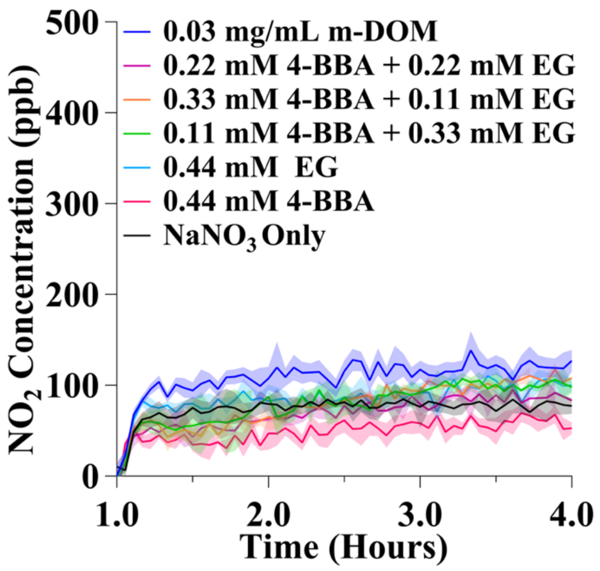

Figure S6. The NO_2 concentration measured in ppb during the irradiation period of the experiments (t = 1 – 4 hours) for all solutions containing acidified 100 mM $NaNO_3$ with mixtures of the molecular proxies 4-BBA and EG, the molecular proxies on their own, and 0.03 mg/mL m-DOM.

Extraction of m-DOM from SeaSCAPE campaign

The extraction of m-DOM during the 2019-NSF CAICE SeaSCAPE Campaign has been previously described by Alves et al., 2022.[5] The collaborative mesocosm project SeaSCAPE was described in detail by Sauer et al, 2021.[11] Briefly, approximately 11,800 L of seawater was gathered from Ellen Browning Scripps Memorial Pier in La Jolla, California. This seawater was filtered to 50 microns to eliminate non-microbial biota and large grazers. The filtered seawater was then transferred to a cleaned glass wave channel and allowed to stabilize at ambient temperature for 24 hours. Following this, nutrients were introduced into the wave channel with fluorescent lights to replicate a diurnal light cycle. Over the 23-day period of microbial bloom development, samples were collected, from which dissolved organic matter (DOM) was isolated through sequential filtration. Post-filtration, the inorganic carbon was removed by acidification to pH 2.00 using 1M HPLC-grade HCl. The resulting DOM was then purified via solid-phase extraction. Specifically for this study, the DOM used was the DOM acquired from filtering the entirety of the seawater used at the end of the campaign.

Replicate Experiments

The limited amount of m-DOM from the 2019-NSF CAICE SeaSCAPE Campaign posed constraints on the number of trials that could be done for this study. The number of trials for each experimental data point are listed in Table S1. Though 3 out of the 8 data points presented are only single experiments, they were included in the discussion as the trends m-DOM concentrations had on HONO enhancement were clear and supported by the single experiments.

Table S1. The number of replicate experiments each experiment containing m-DOM

Amount of m-DOM (mg/mL)	Number of Replicate Experiments
0.00	3
0.01	1
0.03	1
0.05	3
0.10	3
0.20	2
0.40	1
0.60	2

Surface Tension of Molecular Proxies

Table S2. Surface tension of solutions all containing 100 mM $NaNO_3$ and acidified to pH 2.0 with HCl. The uncertainty represents one standard deviation of triplicate experiments.

Solutions Containing 100 mM $NaNO_3$ at pH 2.0	Surface Tension (mN/m)
No organics present	73.8 ± 0.1
0.03 mg/mL m-DOM	70.0 ± 0.5
0.44 mM 4-BBA	72.3 ± 0.2
0.44 mM EG	73.1 ± 0.1
0.11 mM 4-BBA + 0.33 mM EG	73.5 ± 0.2
0.22 mM 4-BBA + 0.22 mM EG	74.6 ± 1.7
0.33 mM 4-BBA + 0.11 mM EG	72.9 ± 0.4

References

(1) Kraus, S. DOASIS: A Framework Design for DOAS, University of Mannheim Germany, 2006.

(2) Stutz, J.; Kim, E. S.; Platt, U.; Bruno, P.; Perrino, C.; Febo, A. UV-Visible Absorption Cross Sections of Nitrous Acid. *J. Geophys. Res. Atmos.* **2000**, *105* (D11), 14585–14592.

(3) Burrows, J. P.; Dehn, A.; Deters, B.; Himmelmann, S.; Richter, A.; Voigt, S.; Orphal, J. Atmospheric Remote-Sensing Reference Data from GOME: Part 1. Temperature-Dependent Absorption Cross-Sections of NO_2 in the 231-794 nm Range. *J. Quant. Spectrosc. Radiat. Transf.* **1998**, *60*, 1025–1031.

(4) Karimova, N. V.; Alves, M. R.; Luo, M.; Grassian, V. H.; Gerber, R. B. Toward a Microscopic Model of Light Absorbing Dissolved Organic Compounds in Aqueous Environments: Theoretical and Experimental Study. *Phys. Chem. Chem. Phys.* **2021**, *23*, 10487–10497.

(5) Alves, M. R.; Coward, E. K.; Gonzales, D.; Sauer, J. S.; Mayer, K. J.; Prather, K. A.; Grassian, V. H. Changes in Light Absorption and Composition of Chromophoric Marine-Dissolved Organic Matter across a Microbial Bloom. *Environ. Sci.: Process Impacts* **2022**, *24*, 1923–1933.

(6) Karimova, N.; Alija, O.; Mora García, S. L.; Grassian, V. H.; Gerber, R. B.; Navea, J. G. pH Dependence of the Speciation and Optical Properties of 4-Benzoylbenzoic Acid. *Phys. Chem. Chem. Phys.* **2023**, *25*, 17306–17319.

(7) Zhang, J.; Zhang, P.; Ma, K.; Han, F.; Chen, G.; Wei, X. Hydrogen Bonding Interactions between Ethylene Glycol and Water: Density, Excess Molar Volume, and Spectral Study. *Sci. China. B Chem.* **2008**, *51*, 420–426.

(8) Reeser, D. I.; Kwamena, N. O. A.; Donaldson, D. J. Effect of Organic Coatings on Gas-Phase Nitrogen Dioxide Production from Aqueous Nitrate Photolysis. *J. Phys. Chem. C* **2013**, *117*, 22260–22267.

(9) *NIST Chemistry WebBook Nitrogen Dioxide (Accessed February 2023). https://webbook.nist.gov/cgi/cbook.cgi?ID=C10102440&Mask=10#Solubility.*

(10) *NIST Chemistry WebBook Nitrous Acid (Accessed February 2023). https://webbook.nist.gov/cgi/cbook.cgi?ID=C7782776&Mask=10#Solubility.*

(11) Sauer, J. S.; Mayer, K. J.; Lee, C.; Alves, M. R.; Amiri, S.; Bahaveolos, C.; Barnes, E. B.; Crocker, D. R.; Dinasquet, J.; Garofalo, L. A.; Kaluarachchi, C. P.; Dang, D.; Kilgour, D.; Mael, L.; Mitts, B. A.; Moon, D. R.; Morris, C. K.; Moore, A. N.; Ni, C.-M.; Pendergraft, M. A.; Petras, D.; Simpson, R.; Smith, S.; Tumminello, P. R.; Walker, J. L.; DeMott, P. J.; Farmer, D. K.; Goldstein, A. H.; Grassian, V. H.; Jaffe, J. S.; Malfatti, F.; Martz, T. R.; Slade, J.; Tivanski, A. V.; Bertram, T. H.; Cappa, C. D.; Prather, K. A. The Sea Spray Chemistry and Particle Evolution Study (SeaSCAPE): Overview and Experimental Methods. Environ. Sci.: Process Impacts **2022**, *24*, 290–315.
